# Knowledge, attitudes and practices of hepatitis B prevention and immunization of pregnant women and mothers in northern Vietnam

**DOI:** 10.1371/journal.pone.0208154

**Published:** 2019-04-10

**Authors:** Thi T. Hang Pham, Thuy X. Le, Dong T. Nguyen, Chau M. Luu, Bac D. Truong, Phu D. Tran, Mehlika Toy, Samuel So

**Affiliations:** 1 Asian Liver Center, Stanford University School of Medicine, Palo Alto, California, United States of America; 2 General Department of Preventive Medicine, Ministry of Health, Hanoi, Vietnam; University of Washington, UNITED STATES

## Abstract

**Background and aim:**

Infection at birth due to mother-to-child (MTC) transmission is the most common cause of chronic hepatitis B virus (HBV) infection in Vietnam. This study was undertaken to examine the knowledge, attitudes, and practices of pregnant women and mothers in Vietnam concerning HBV prevention and immunization.

**Methods:**

A cross-sectional survey was conducted in Quang Ninh and Hoa Binh provinces in 2017. A standardized questionnaire was administered to women when they received care at primary and tertiary maternal health clinics. Multivariate regression was used to identify predictors of HBV knowledge and practices.

**Results:**

Among the 380 women surveyed, 50.3% were pregnant and 49.7% were postpartum. Despite 70.3% of participants reported having received information about HBV during their pregnancy, only 10.8% provided correct answers to all questions regarding HBV transmission routes and preventive measures. Around half of the participants incorrectly believed that HBV is transmitted through sneezing, contaminated water or sharing foods with chronic HBV patients. Although 86.1% of participants believed that HBV vaccination is necessary for infants, only 66.1% responded they were definitely willing to have their own child vaccinated within 24 hours. More than a third of participants expressed concern about having casual contacts or sharing foods with chronic HBV patients. In multivariate analysis, having received information about HBV during their pregnancy was significantly associated with better HBV knowledge score. Delivery at provincial level clinics was a strong predictor for perinatal HBV screening and hepatitis B birth dose administration.

**Conclusions:**

The results highlight the need to prioritize educating pregnant women and mothers in future public health campaigns in order to increase knowledge, reduce misperception, and improve hepatitis B vaccine birth dose coverage in Vietnam.

## Introduction

Liver cancer is the fourth most common cause of death from cancer and carries the second highest rate of absolute years of life lost among cancers globally in 2016 [1]. The 2016 Global Burden of Disease also estimates that HBV infection alone accounts for about 42% of liver cancer deaths.

Vietnam has the 6th highest incidence of liver cancer and the third highest rate of death from liver cancer in the world in 2018 [2]. In 2018, liver cancer was the third leading cause of cancer death in Vietnam after lung and stomach cancer [2]. HBV accounts for close to half (46%) of the liver cancer deaths in Vietnam [2]. The reported prevalence of the hepatitis B surface antigen (HBsAg) in the general population ranged from 15–20% [3–6]. Current estimates suggests 10.8% of the population or 9.6 million people in Vietnam are HBsAg positive and are living with chronic hepatitis B [4].

The transmission of HBV can be effectively prevented through immunization with the hepatitis B vaccine. Infant hepatitis B vaccination was introduced to Vietnam in 1997 and expanded nationwide in 2002 [5]. To combat the significant risk of infection at birth due to MTC transmission, a birth dose administered within 24 hours after birth was added to the immunization schedule in 2003 [7]. The results of a nationwide survey comparing HBsAg prevalence in children born between 2000–2003 to children born between 2007–2008 showed a 2% reduction in prevalence [5]. Additionally, infants vaccinated > 7 days after birth showed a 1.68% increase in HBsAg prevalence compared to those vaccinated within the 24 hour following the immunization guideline [5]. Such findings demonstrate the profound impact of both implementation and timely birth dose vaccination in reducing the risk of chronic hepatitis B infection. Despite these encouraging results, birth-dose vaccination coverage has struggled to stay consistent since its implementation. Birth dose coverage dropped to its lowest rate in 2010 at 21.4%, rose to its highest in 2012 at 75% before dropping to 56% in 2013 [8]. The reporting of adverse events following immunization (AEFIs) in 2007 and 2013 that were blamed on newborn hepatitis B vaccination in conjunction the emergence of anti-vaccination movements may contribute to this volatility [9, 10]. The impact of a 19% drop in coverage was estimated to increase the burden by 130,675 new chronic HBV infections and 25,197 HBV-associated deaths for children born in 2013 [9].

As perinatal transmission continues to be the major route of transmission in Vietnam, it is critical to initiate strategies to engage women of reproductive age to prevent mother-to-child transmission. Such strategies would require mothers to understand the necessity of HBV testing during pregnancy, the benefits of timely infant hepatitis B vaccination, and for infected mothers the importance of the newborn receiving hepatitis B birth dose and hepatitis B immune globulin within 24 hours after birth and completing the vaccine series. A number of studies have found that knowledge about HBV, including transmission routes and prevention, was poor among pregnant women [11–16]. Several studies showed more than half of the pregnant women did not know HBV can be transmitted by unprotected sex [10–12]. To our knowledge, there is currently little data regarding HBV knowledge, attitudes, and practices among Vietnamese pregnant women and mothers.

The Northern region of Vietnam is home to a third of the national population [17]. Reported birth dose coverage was only 55.4% in 2014 [8]. This study assesses the knowledge, attitudes, and practices of pregnant women and mothers in the region concerning HBV prevention and immunization. The findings from this survey will help to develop targeted public health intervention to prevent mother-to-child transmission of hepatitis B and to improve birth dose coverage.

## Methods

### Study population

This cross sectional study was undertaken from February through August 2017. Out of 25 Northern provinces, two provinces were selected representing the Northern Midlands and Mountains (Hoa Binh) and the Red River Delta (Quang Ninh). Among the 102 facilities that provided maternal care to at least 150 patients per year, two provincial hospitals, 6 district health clinics and 8 commune health centers were selected for this study. All women who presented at the clinics for regular prenatal care, delivery or postpartum check-up within 60 days of delivery were eligible. Trained data collectors administered a pre-designed questionnaire at maternal care clinics. Study protocol and ethical approval was obtained from the Scientific Research Committee of The Vietnam Family Planning Association (reference number: 052017/HD5). Written consent form was obtained from the participants before the interview.

The sample size was determined by using a single population proportion formula considering the following assumptions: the prevalence of women who have correct knowledge about hepatitis B prevention and immunization is 50%, a confidence interval of 95%, and a margin of error of 5%. The sample size was calculated to be 380. After factoring in an assumed non-response rate of 8%, the final sample size was 410. Number of study participants recruited for each clinic was proportional to its number of births per year.

### Questionnaire

The questionnaire was developed in Vietnamese by the Asian Liver Center at Stanford University based on its past experience with administering HBV knowledge surveys in other populations [18, 19] and a literature review of comparable studies [11, 12, 14, 20]. The questionnaire was pre-tested on 20 subjects to check the language, flow and comprehension of the questions. After the pre-test, a few modifications were made to assure the questions were comprehensible and interpreted as intended. The length of each interview was approximately 20 minutes. The questionnaire consisted of four sections: i) demographic and personal HBV-related health history; ii) disease burden and consequences; iii) transmission routes and prevention measures; and iv) for postpartum mothers, when the newborn received the hepatitis B vaccine after birth. There were 18 questions on HBV knowledge (questions 9,10, 12–24, 26, 28, 31), five questions on HBV attitudes (questions 29, 30, 34, 35, 36), and two questions on antenatal screening and newborn vaccination practices (questions 25,39) (See S1 Text).

Ten research assistants who were not affiliated with the selected health clinics were trained by the Vietnam General Department of Preventive Medicine to administer the surveys. The participants were assured that the information collected will be kept confidential and the study does not collect personal or identifiable data. After explaining the survey’s background, purposes, procedures and confidentiality, only those who gave a written consent were included in the study. The participants received no compensation. Completed questionnaires were safely kept in locked cabinets and was only accessed by the study investigators.

### Statistical methods

The STATA 12.0 statistical software was used for analysis. Descriptive statistics were generated from variables in the dataset.

HBV knowledge score was calculated based on the sum of correct answers to the 18 knowledge questions. A correct response to each question received one point and incorrect or missing responses received no points. First, univariate linear regression was performed to measure the association between HBV knowledge score and demographic factors (i.e., age, gender, occupation, education, family income), maternal factors (number of children, gestational age, type of facility where mother received maternal care), and whether the mother received HBV information during pregnancy. Variables with a p value <0.25 in univariate were included into multivariate analysis [21]. Co-efficient and 95% confidence intervals (CIs) were used when measuring the association of the different characteristics with the outcomes of interest. Degree of statistical significance was declared at a p value ≤ 0.05

HBV antenatal screening uptake and hepatitis B birth dose administration were used as the primary practice outcome variables. Univariate logistic regression was performed to evaluate the strengths of the association between outcome variables with several explanatory variables (age, occupation, education level, income, number of children, type of health facility where the mother gave birth, and HBV knowledge score). Variables with a p value <0.25 in univariate were included into multivariate analysis. Odds ratios (ORs) and 95% confidence intervals (CIs) were used when measuring the association of the different characteristics with the outcomes of interest. Degree of statistical significance was declared at a p value ≤ 0.05.

## Results

### Demographics of survey participants

Of the 410 pregnant women who were approached, 404 agreed to participate with a response rate of 98.5%. Among the 404 participants, 24 did not complete the survey questions and were excluded from the study analysis. Demographics and maternal characteristics of the study population (N = 380) are presented in Table 1. Among the women surveyed, 57.4% visited the clinics for prenatal care or delivery and 42.6% visited the clinics for postpartum care. 189 of them (49.7%) gave birth within the past 1–60 days. Participants’ age range from 17 to 45 years (median age: 27 years, mean age: 27 years). 74.2% completed high school level education or higher. The most common occupations were farming (23.4%), housewife (22.4%), and clerk or administrative positions (20.3%). Around 14.7% had an household income below the national poverty line for rural and mountainous areas (defined as <400,000 Vietnam Dong or 17 USD per capita each month), compared to the average poverty rate at 11.8% for Northern midlands and mountain areas in 2016 [22]. Location of antenatal care was primarily at province level hospitals (47.6%) with the remaining approximately evenly split between district (26.3%) and commune health facilities (21.6%).

**Table 1:** Demographics of survey participants (N = 380 pregnant/postpartum women).

Respondent’s Demographics		N		(%)	
**Age**		***380***		***100***	
Under 25 years		104		27.4	
25–34 years		242		63.7	
≥ 35 years		34		8.9	
**Employment**		***380***		***100***	
Farming		89		23.4	
Small trade business		47		12.4	
Housewife		85		22.4	
Clerk/admin		77		20.3	
Teacher		40		10.5	
Others		42		11.1	
**Education Level**		***380***		***100***	
None—Secondary		98		25.8	
High School		129		33.9	
College–University or Higher		153		40.3	
**Average per capita income in VND each month**		***380***		***100***	
< 400,000 (below poverty line)		56		14.7	
≥ 400,000		324		85.3	
**Pregnancy Status**		***380***		***100***	
First trimester (1–3 month)		65		17.1	
Second trimester (4–6 month)		64		16.8	
Third trimester to delivery (7 month- delivery)		89		23.4	
Postpartum visit		162		42.6	
**Facility where mother received care**		***380***		***100***	
Commune health center		82		21.6	
District health hospital		100		26.3	
Province level hospital		181		47.6	
Others (including private clinic)		17		4.5	
**Number of children**		***380***		***100***	
0		57		15.0	
1		180		43.4	
> = 2		143		37.6	
**Mothers received information about HBV prevention during this pregnancy**		***380***		***100***	
Yes		267		70.3	
No		96		25.3	
Don’t remember		17		4.4	
**Mothers received information about the benefits of hepatitis B vaccine for infants**		***380***		***100***	
Yes		330		86.8	
No		50		13.2	
**If mothers received information about the benefit of hepatitis B vaccine for infants, what were the sources? (N = 330)**		***330***		***100***	
Advice from health care workers		300		90.9	
Flyers		115		34.8	
Newspapers and magazines		120		36.4	
Radio programs		107		32.4	
Television programs		127		38.5	
Internet		124		37.6	
Other		9		2.7	

About 70.3% of participants reported that they received information about HBV during their pregnancy. Around 86.8% reported that they previously received information about the benefit of hepatitis B vaccine for infants. Healthcare workers were the primary source of information about benefits of hepatitis B vaccine (90.9%) followed by equal contribution from common public outreach methods such as flyers, newspapers, radio, television, and the internet.

### Knowledge about HBV and Prevention

Out of 18 HBV questions, the mean knowledge score was 12.05 ± 3.37 (mean ± SD) and the median was 12 (interquartile range (IRQ) 10–15). Only 10.8% of study participants provided correct answers to all 12 questions on HBV transmission modes and preventive measures. 36.1% provided correct answers to all 4 questions regarding prevention of mother-to-child prevention.

Only 25.8% were aware of the high prevalence of chronic hepatitis B infection in Vietnam. Only 57.9% of participants were aware that chronic HBV can cause serious consequences such as liver cirrhosis, liver failure, liver cancer, or premature death. Study participants were largely aware that HBV can be transmitted through mother-to-child (84.2%), unprotected sex (75.3%), and blood transfusions (85.8%). However, there were common misconceptions that HBV can be transmitted through sneezing or coughing (41.8%), contaminated water (45.8%), and eating with or sharing food with chronic HBV patients (52.4%) (Table 2).

**Table 2:** Knowledge about HBV and Prevention (N = 380 pregnant/postpartum women).

Questions	Correct answers	
	N	%
**Prevalence and Sequelae of Infection**		
Q9. How many percent of Vietnam population has chronic HBV?	98	25.8
Q. 10 What can chronic HBV infection cause?	220	57.9
**Transmission Routes**		
Q12. Hepatitis B can be transmitted through handshake	329	86.6
Q13. Hepatitis B can be transmitted through contaminated water	206	54.2
Q14. Hepatitis B can be transmitted through unprotected sex	286	75.3
Q15. Hepatitis B can be transmitted through blood transfusion	326	85.8
Q16. Hepatitis B can be transmitted through sneezing or coughing	221	58.2
Q17. Hepatitis B can be transmitted through from mother to her child at birth	320	84.2
Q18. Hepatitis B can be transmitted through eating with or sharing food/utensils	181	47.6
**Prevention Measures**		
Q19. Clean and cook food thoroughly can prevent HBV transmission	109	28.7
Q20. HBV vaccination can prevent HBV transmission	352	92.6
Q21. Do not reuse or share needles/syringes can prevent HBV transmission	342	90.0
Q22. Avoid sharing food/utensils or eating with a person with chronic HBV can prevent HBV transmission	153	40.3
Q23. Use a condom can prevent HBV transmission	295	77.6
**Mother-to-Child Prevention**		
Q24. As a pregnant woman, do you need to be tested for HBV?	312	82.1
Q26. Is HBV vaccination necessary for your infant?	327	86.1
Q28. When is best time to provide a healthy and stable child the first dose of HBV vaccine?	304	80.0
Q31. If a pregnant woman has chronic HBV, what measure could protect the newborn from becoming infected?	197	51.84

The percentage of participants who were aware that HBV can be prevented by receiving the hepatitis B vaccine, not reusing or share needle/syringes and using condom were 92.6%, 90.0% and 77.6% respectively. 71.3% had the misconception that cooking and cleaning of food can prevent HBV transmission. 59.7% thought avoiding sharing food and utensils or eating with person with chronic HBV can prevent HBV transmission (Table 2)

### Attitude towards HBV

About a third of the surveyed pregnant women and mothers had concerns about having casual contact (31.8%), working with or sharing food with chronic HBV patients (37.4%). Moreover, 40.8% of respondents expressed having concerns if their child was in the same class with a child with chronic HBV infection. While most participants were aware that infant hepatitis B vaccination is necessary (86.1%) and the best time to provide a healthy and stable child the first dose of HBV vaccine is within 24 hours after birth (80.0%) (Table 2), their confidence in giving their own children the hepatitis B vaccine birth dose was lower. Only 66.1% of mothers responded that they would definitely be willing to have their own child vaccinated within 24 hours even if their doctors tell them the vaccine is safe (Table 3).

**Table 3:** Attitudes towards HBV transmission (N = 380 pregnant/postnatal women).

Questions	N	%
**Would you have any concern having casual contact or working together with a CHB patient in the same office?**		
Yes	121	31.8
No	206	54.2
Not sure	53	14.0
**Would you have any concern eating with (sharing food or utensils) with a CHB patient?**		
Yes	142	37.4
No	187	49.2
Not sure	51	13.4
**Would you have any concern if your child is in the same class with a child that is infected with HBV?**		
Yes	155	40.8
No	186	49.0
Not sure	39	10.2
**If your newborn is healthy and stable, would you think it is safe to give your baby the HBV vaccine in the first 24 hours after birth?**		
Very safe	277	72.9
Maybe safe	77	20.3
Not very safe	26	6.8
**If your doctor tells you that HBV vaccine is safe to given to a newborn, would you be willing to provide your child the HBV vaccine?**		
Definitely	251	66.1
Maybe	102	26.8
Not sure	23	6.0
No	4	1.1

### Factors associated with HBV knowledge

In multivariate linear regression analysis, having received information about HBV during pregnancy was the only factor independently associated with HBV knowledge score after controlling for other variables. Age, occupation, education level, number of children, household income and type of health facilities where mothers received maternal care were not associated with HBV knowledge score (Table 4).

**Table 4:** Analysis of factors associated HBV knowledge scores (380 pregnant/postnatal women).

	Univariate linear regression analysis			Multivariate linear regression analysis		
Variables	Coef.	t	P	Adjusted Coef.	95% CI	P
**Age (re: 17–24 years)**						
25–34	-0.002	-0.01	0.10	-0.10	-0.85 to 0.66	0.81
> = 35 years	0.01	0.02	0.99	0.04	-1.24 to 1.24	0.96
**Occupation (re: farming)**						
Small trade business	0.50	0.82	0.41	0.03	-1.20 to 1.23	0.97
Housewife	0.72	1.42	0.16	0.24	-0.84 to 1.21	0.65
Clerk/admin	1.30	2.47	0.14	0.78	-0.28 to 1.93	0.16
Teacher	1.03	1.61	0.11	0.53	-0.68 to 1.92	0.41
Others	0.54	0.85	0.39	0.30	-0.96 to 1.47	0.63
**Education (re: middle school and lower)**						
High school	1.04	0.23	0.82			
College or higher	0.46	1.05	0.29			
**Family income below poverty line (re: yes)**						
No	1.18	0.24	0.81			
**Number of children (re: 0)**						
1	-0.08	-0.15	0.88			
> = 2	-0.57	-1.08	0.28			
**Pregnancy status at the time of interview (re: first trimester)**						
Second trimester	0.70	1.18	0.24	0.46	-0.66 to 1.65	0.42
Third trimester—delivery	-0.10	-0.17	0.86	-0.30	-1.31 to 0.74	0.56
Postnatal mothers	0.43	0.86	0.39	0.15	-0.74 to 1.19	0.75
**Facility where mother received maternal care (re: commune health center)**						
District health hospital	0.51	1.02	0.31	0.72	-0.33 to 1.66	0.15
Province level hospital	0.64	1.44	0.15	0.51	-0.49 to 1.41	0.28
Others	0.41	0.46	0.65	0.47	-1.31 to 2.22	0.60
**Mother receiving HBV education during pregnancy (re: yes)**						
No	-2.81	-7.53	0.000	-2.75	-3.51 to -1.98	<0.001
Don’t remember	-2.59	-3.29	0.001	-2.52	-4.21 to -1.02	0.002

### HBV screening and immunization practice

In the subgroup of 189 postpartum women surveyed, 118 (62.4%) reported receiving hepatitis B testing during their most recent pregnancy. Among them, 17.8% reported having positive results and 10.2% were unsure of their results. 65.6% reported their newborn were administered the first dose of the hepatitis B vaccine within 24 hours of birth. 12.7% were vaccinated between 24 to 48 hours after birth and 13.7% did not receive any vaccine until 1 month of age. When asked why the infants were not vaccinated within 24 hours of birth, the following responses were given: mother did not think it was safe (28%); no vaccine available (20%); child was sick (18%); mother did not think it was necessary (14); doctor said it was not necessary (10%) and child has low birth weight (4%) (Table 5).

**Table 5:** HBV antenatal screening and hepatitis B birth dose vaccination practices (N = 189 postpartum women).

Question	N	(%)
**Were you tested for hepatitis B during the most recent pregnancy? (N = 189)**	**189**	**100**
Yes	118	62.4
No	58	30.7
Don’t remember	13	6.9
**If yes, were you given the test results? (N = 118)**	**118**	**100**
Positive	21	17.8
Negative	85	72.0
Don’t know	12	10.2
**When did your baby receive the first dose of HBV vaccine? (N = 189)**	**189**	**100**
Within 24 hours after birth	124	65.6
Between 24–48 hours after birth	24	12.7
Did not receive any vaccine until 1 month of age	26	13.7
Don’t know	15	7.9
**What was the reason for not giving the baby an HBV vaccine within 24 hours after birth? (N = 50)**	**50**	**100**
Mother did not think it was necessary	7	14.0
Mother did not think it was safe	14	28.0
Mother did not understand why it was not given	3	6.0
No vaccine available	10	20.0
Child was sick	9	18.0
Child had a low birth weight	2	4.0
Doctor said it was not necessary	5	10.0

### Factors associated with HBV antenatal screening and hepatitis B vaccine birth dose administration

In multivariate logistic regression analysis, giving birth at province level hospitals was independently associated with both maternal antenatal HBV screening uptake and newborns receiving the hepatitis B birth dose. The adjusted ORs associated with HBV antenatal screening uptake and with administration of hepatitis B birth dose were 6.61 (95% CI: 2.04–21.45) and 4.39 (95% CI: 1.48–13.02) respectively (Table 6 and Table 7). Mothers receiving information about the benefits of newborn hepatitis B vaccination was also independently associated with the newborn receiving hepatitis B birth dose. Age, occupation, education level, number of children, household income and HBV knowledge score were not associated with whether the newborns received the hepatitis B birth dose or not (Table 7).

**Table 6:** Analysis of factors associated with antenatal HBV screening uptake (N = 189 postpartum women).

Variables	Univariate logistic regression analysis			Multivariate logistic regression analysis		
	OR	95% CI	P	AOR	95% CI	P
**Age (years) (re: 17 - <25)**						
25–34 years	1.74	0.82 to 3.66	0.15	1.46	0.59 to 3.62	0.41
> = 35	2.31	0.70 to 7.65	0.17	1.68	0.38 to 7.37	0.49
**Occupation (re: farming)**						
Small trade business	5.40	1.67 to 17.50	0.005	2.96	0.75 to 11.62	0.12
Housewife	2.03	0.80 to 5.19	0.137	1.47	0.479 to 4.43	0.49
Clerk/admin	7.93	2.84 to 22.12	0.000	2.24	0.64 to 7.87	0.21
Teacher	8.50	2.14 to 33.81	0.002	6.24	1.25 to 31.25	0.03
Others	3.30	0.96 to 11.31	0.058	1.89	0.46 to 7.73	0.38
**Education (re: middle school and lower)**						
High school	0.45	0.19 to 1.02	0.06	0.77	0.27 to 2.21	0.62
College or higher	1.26	0.57 to 2.78	0.56	2.01	0.75 to 5.39	0.16
**Family income below poverty line (re: yes)**						
No	1.43	0.60 to 3.41	0.42			
**Number of children (re: 1)**						
> = 2	1.18	0.63 to 2.22	0.60			
**Health facility where delivery was taking place (ref: commune health center)**						
District health hospital	1.70	0.64 to 4.57	0.28	1.45	0.48 to 4.42	0.51
Province level hospital	9.33	3.51 to 24.77	<0.001	6.61	2.04 to 21.45	0.002
**Total HBV knowledge score (continuous variable)**	1.12	1.01 to 1.24	0.03	1.11	0.98 to1.25	0.90

**Table 7:** Analysis of factors associated with newborn receiving the hepatitis B vaccine birth dose (N = 189 postpartum women).

Variables	Univariate logistic regression			Multivariate logistic regression		
	OR	95% CI	P	AOR	95% CI	P
**Age (years) (re: 17 - <25)**						
25–34 years	1.46	0.73 to 2.93	0.29			
> = 35	1.64	0.54 to 5.04	0.39			
**Occupation (re: farming)**						
Small trade business	3.11	1.00 to 9.67	0.05	1.35	0.37 to 4.89	0.65
Housewife	1.22	0.51 to 2.95	0.65	0.95	0.34 to 2.64	0.92
Clerk/admin	1.85	0.78 to 4.40	0.16	0.83	0.26 to 2.63	0.75
Teacher	3.11	1.00 to 9.67	0.05	1.35	0.37 to 4.89	0.65
Others	1.71	0.51to 5.69	0.38	0.90	0.22 to 3.70	0.89
**Education (re: middle school and lower)**						
High school	1.41	0.64 to 3.10	0.39	1.85	0.68 to 5.03	0.23
College or higher	1.68	0.81 to 3.49	0.17	1.84	0.77 to 4.39	0.17
**Family income below poverty line (re: yes)**						
No	1.76	0.82 to 3.79	0.14	1.34	0.56 to 3.22	0.51
**Number of children (re: 1)**						
> = 2	1.71	0.93 to 3.14	0.08	1.46	0.74 to 2.87	0.27
**Health facility where delivery was taking place (ref: commune health center)**						
District health hospital	1.40	0.47 to 4.45	0.51	1.31	0.47 to 3.62	0.60
Province level hospital	4.69	1.87 to 11.75	0.001	4.39	1.48 to 13.02	0.008
**Mother receiving information about benefits of hepatitis B vaccine during pregnancy (re: Yes)**						
No	0.27	0.11 to 0.63	0.003	0.34	0.12 to 0.88	0.026
**Total HBV knowledge score (continuous variable)**	1.07	0.97 to 1.12	0.16	1.07	0.96 to 1.19	0.24

## Discussion

This survey showed that pregnant women and mothers in Northern Vietnam’s Quang Ninh and Hoa Binh provinces lacked knowledge regarding HBV transmission and prevention regardless of age, education, occupation, household income and prior exposure to HBV information. Limited knowledge regarding HBV among pregnant women is consistent with findings from previous studies in other high epidemic countries [11–15, 20]. When compared to previous studies in endemic countries, participants in this survey seemed to be more knowledgeable about HBV transmission routes. For instance, 75.3% of participants in this study were aware that HBV is transmitted through unprotected sex compared to 46.7% to 52.9% in China [11, 12], and 41.2% in Nigeria [13]. About 85.8% of study participants recognized that HBV can be transmitted through blood transfusion compared to 46.7% to 75.5% in China [11, 12], and 10.3% in Nigeria [13]. It is noteworthy that information related to HBV and hepatitis B vaccine was relatively accessible to pregnant women at the study sites. Most of the study participants reported receiving information about HBV prevention (70.3%) and benefits of hepatitis B vaccine for infants (80.6%) during the current or most recent pregnancy. Statistical analysis also showed that exposure to information during pregnancy has a positive impact on HBV knowledge score as well as on whether the newborn received the hepatitis B birth dose. Our findings emphasize a need to improve education programs targeting women of childbearing age about HBV and the benefits of hepatitis B antenatal testing and hepatitis B vaccine birth dose. It is also necessary to review existing antenatal educational programs and materials to ensure that key messages are effectively conveyed to the target audiences.

Universal hepatitis B testing of pregnant women in antenatal clinics is strongly recommended by the World Health Organization to reduce mother-to-child transmission of HBV [23]. In Vietnam, however, HBV screening is not yet considered a routine test in prenatal care package. While HBV perinatal screening has been implemented in many central and provincial hospitals, there is lack of data on HBV perinatal screening coverage. The Vietnam National Action Plan to Eliminate HIV, HBV and Syphilis for the period from 2018–2030 aims to test at least 50% of pregnant women during the 2018–2020 period and at least 70% of pregnant women during the 2020–2025 period for HBV [24]. In this study, 62.4% of study participants reported being screened for HBV during the most recent pregnancy. Multivariate regression results also showed that giving birth at provincial health clinics is a strong predictor of HBV antenatal screening. As such, the study results emphasizes the need to have targeted interventions to improve HBV perinatal screening for pregnant women, particularly at primary healthcare levels.

Low hepatitis B birth dose coverage presents a serious threat to prevention of mother-to-child transmission of HBV because delayed administration of hepatitis B vaccine birth dose can significantly reduce its effectiveness. Missed hepatitis B vaccine birth dose is also a risk factor for incomplete vaccination later in childhood [25]. In Vietnam, the national birth dose coverage was reported at 68% and 77% for 2016 and 2017 respectively [26]. Other studies reported birth dose coverage of only 46.6% in the Mekong River Delta [27] and 59.1% in the northern mountainous region [28]. Finding from this study indicated that birth dose coverage has not been fully restored in the study region with only 65.6% of postnatal mothers reported their newborn received the first dose of the hepatitis B vaccine within 24 hours after birth. One explanation for the low birth dose practice was the low confidence in the vaccine birth dose safety among pregnant women. In this study, only 66.1% of participants expressed willingness to have their own child vaccinated within 24 hours after birth even when the doctors say that the vaccine is safe. This was lower compared to a study in China in which 89.9% of pregnant women were willing to let their newborn receive the hepatitis B birth dose [10]. Concerns over the hepatitis B vaccine safety was also the primary reasons (28.0%) given by mothers for not giving the newborn HBV birth dose. Our multivariate analysis suggests giving birth at province level hospitals was a strong predictor for receiving the hepatitis B birth dose. This might be because HBV vaccine is more available, health care workers are more competent, or quality control is better implemented at higher level of care to facilitate birth dose administration. In this study, the other reasons birth dose was not given were vaccine unavailability (20% of the cases), and “doctors said it is not necessary” (10% of cases) suggesting the need to also educate healthcare workers in addition to pregnant women. Taken together, these data underscore a critical need to have a more complete understanding of barriers to hepatitis B vaccine birth dose practice to inform public health solutions on how best to restore and improve birth dose coverage.

This study also revealed significant stigma associated with people having chronic HBV in Vietnam. About a third of participants expressed concerned about eating with, sharing food, casual contact or working in the same office with chronic HBV patients. A higher proportion (40.8%) expressed concerns if their children were in the same class with a child with chronic HBV infection. One explanation could be because approximately half of the women surveyed believed that HBV can be transmitted through sneezing or coughing, contaminated food and water, or eating or sharing food with chronic HBV patients. The stigma and pattern of knowledge deficits observed in this study regarding HBV was similar to a previous study among adult residents in Ho Chi Minh city in which 55% had the mistaken impression that HBV can be spread by sharing eating utensils and 61% felt that persons with chronic HBV infection put others at risk [29]. It is widely recognized that HBV related stigma can negatively affect health behaviors related to screening, prevention, diagnosis and treatment for HBV infection [30]. Further research into this area to understand the magnitude, underlying reasons and its impact is necessary to evaluate effective interventions to improve awareness and tackle stigma in HBV in Vietnam.

## Study limitations

This study was conducted in two Northern provinces of Vietnam with low HBV birth dose coverage at the study point; thus, the results may not be applicable to other parts of the country. In addition, the self-reported HBV screening and immunization practices by study participants could not be validated.

## Conclusion

This study shows that pregnant women and mothers have insufficient knowledge and practice regarding HBV infection regardless of age, education, socio-economic status, childbearing status and prior exposure to HBV information during pregnancy. Misconceptions about HBV transmission through contaminated water, sharing foods and casual contacts were common and perpetuated the stigma associated with chronic HBV infection. Although most participants were aware of the benefits of hepatitis B vaccine, concerns about vaccine safety for newborn were prevalent. This emphasizes the need to enhance public health education efforts to improve hepatitis B knowledge among women in reproductive age and demystify issues surrounding HBV transmission and vaccine safety to improve hepatitis B birth dose vaccination rate and eliminate mother to child transmission. Public health interventions to improve HBV antenatal screening and hepatitis B birth dose practices are needed, particularly at primary healthcare settings, to eliminate mother-to-child transmission.

## Supporting information

S1 Text (DOCX)

S1 Dataset (DTA)
