# Supplementary material for: Knowledge, attitudes and practices of hepatitis B prevention and immunization of pregnant women and mothers in northern Vietnam
Source: PLoS One. 2019 Apr 10;14(4):e0208154. doi: 10.1371/journal.pone.0208154 (PMC6457574; doi:10.1371/journal.pone.0208154)
Supplement: S1 Text — (DOCX) [file pone.0208154.s001.docx]

**PREGNANT AND POSTPATUM MOTHER SURVEY**

**Patient ID:_____**

**DEMOGRAPHICS**

1. **Current age (years):** _____________
2. **Employment (*Please check ONE*):**

Farming

Small trade business

Housewife

Clerk/admin

Professional

Other (specify): _____________

1. **Education level (*Please check ONE*):**

Non

Elementary

Secondary

High school

College

University or higher

1. **Average family income in million VND each month (Vietnam Dong):** _____________
2. **Number of people in household:** _____
3. **Number of children:** _____
4. **Purpose of this visit (*Please check ONE*):**

First trimester check-up(1-3 month)

Second trimester check-up (4-6 month)

Third trimester or delivery (7month-delivery)

Postpartum visit

1. **Facility where mother is receiving care (*Please check ONE*):**

Commune Health Center

District Health Hospital

Province-level Hospitals

Other (please specify): _____________

**PREVALENCE AND CONSEQUENCESL**

1. **In your opinion, approximately how many percent of Vietnam population has chronic hepatitis B infection? (*Please check ONE*):**
2. 1% (1 in 100)
3. 5% (1 in 20 person)
4. 12% (1 in 8 person)
5. 33% (1 in 3 person)
6. **What can chronic HBV infection cause? (*Please check ONE*):**
7. Liver cirrhosis
8. Liver failure
9. Liver cancer
10. Premature death
11. All of the above
12. Don’t know
13. **11 During your pregnancy, did you receive any information about?**
14. **HIV □** Yes  **□** No
15. **Syphilis □**Yes  **□** No
16. **Hepatitis B □**Yes  **□** No

**TRANSMISION AND PREVENTION**

**Question 12-18: To your understanding, how could hepatitis B be transmitted?**

1. **Shaking hands with a person with chronic HBV**
2. True
3. False
4. Don’t know
5. **Through contaminated water**
6. True
7. False
8. Don’t know
9. **Having unprotected sex with a person with chronic HBV**
10. True
11. False
12. Don’t know
13. **Having blood transfusion**
14. True
15. False
16. Don’t know
17. **Sneezing or coughing**
18. True
19. False
20. Don’t know
21. **From mother with chronic HBV to her child at birth**
22. True
23. False
24. Don’t know
25. **Eating with or sharing food and utensils with a person with chronic HBV**
26. True
27. False
28. Don’t know

**Questions 17-21: What can we do prevent hepatitis B transmission?**

1. **Clean and cook food thoroughly**
2. True
3. False
4. Don’t know
5. **Receive the hepatitis B vaccine**
6. True
7. False
8. Don’t know

1. **Do not reuse or share injection needles/syringes**
2. True
3. False
4. Don’t know
5. **Avoid sharing food and utensils or eating with a person with chronic HBV**
6. True
7. False
8. Don’t know
9. **Use condom**
10. True
11. False
12. Don’t know
13. **As a pregnant woman, do you think that you need to be tested for Hepatitis B? (*Please check ONE*)**
14. Yes
15. No
16. don’t know
17. **Were you tested for hepatitis B during this pregnancy?**
18. Yes
19. No -> go to Q25
20. Don’t remember

**24.1 Were you told whether the test results?**

Positive  Negative  Don’t know

1. **Do you think HBV vaccination is necessary for your infant? (*Please check ONE*)**
2. Yes
3. No
4. Were you told why your baby need HBV vaccine?
5. Yes
6. No
7. **In your opinion, what is the best time to provide a healthy and stable child the first dose of HBV vaccine? (*Please check ONE*)**
8. Within the first 24 hours of birth
9. 1-7 days of birth
10. 1 month old
11. Don’t know
12. **If your newborn is healthy and stable, would you let your baby receive the hepatitis B vaccine in the first 24 hours after birth? (*Please check ONE*):**
13. Very safe -> go to question 29
14. Maybe safe
15. Not very safe
16. **If your doctor tells you that HBV vaccine is safe to given to newborn, will you be willing to get your child HBV vaccine? (*Please check ONE*)**
17. Definitely
18. Maybe
19. Not sure
20. No
21. **If a pregnant woman has chronic hepatitis B, which of the following measure could protect the newborn from becoming infected? (*Please check ONE*)**
22. Administer hepatitis B vaccine to the pregnant woman
23. Administer the first dose of hepatitis B vaccine and the HBIG shot within 12 hours of birth then complete the vaccine series
24. Administer the first dose of hepatitis B vaccine and the HBIG shot within 48 hours of birth then complete the vaccine series
25. **Have you received information about the benefit of hepatitis B vaccine for infant before?**
26. Yes
27. No
28. **If yes, from which of the following sources *(Check all that apply)***
29. Advice from health care workers
30. Flyers
31. Newspapers and magazines
32. Radio programs
33. Television programs
34. Internet
35. Other (specify:____________________________________________________________)
36. **Would you have any concern having casual contact or working together with a chronic HBV patients in the same office? (*Please check ONE*)**
37. Yes
38. No
39. Not sure
40. **Would you have any concern eating with (sharing food or utensils) with a chronic HVBV patient? (*Please check ONE*)**
41. Yes
42. No
43. Not sure
44. **Would you have any concern if you child is in the same class with a chronic HVB kid? (*Please check ONE*)**
45. Yes
46. No
47. Not sure

_____________________________________________________________________________

**This survey ends for pregnant women. Postnatal mothers continue this following part**

**Did you give birth during the last 1-60 days?**

1. **YES 🡪 CONTINUE INTERVIEW**
2. **KHÔNG 🡪 END INTERVIEW**
3. **Where did you give birth? (*Please check ONE*)**
4. Commune health clinic
5. District level clinic
6. Province-level clinic
7. Other (please specify): ……………………………………………………………………………………………………..
8. **What was your baby’s weight at birth? ______kg**
9. **When did your baby receive the first dose of hepatitis B vaccine? (*Please check ONE*)**
10. Within 24 hours after birth
11. Between 24- 48 hours after birth
12. Did not receive any vaccine until 1 month of age
13. Don’t know
14. **Reason for not receiving HBV vaccine 24 hours after birth? (*Please check ONE*):**
15. Don’t think it is necessary
16. There was no vaccine available
17. Child was sick
18. Child was underweight
19. Don’t think it was safe
20. Doctor said it was not necessary
21. It was not offered …………………………………………………………………………………………………………
